# Co-Designing a Text Messaging Intervention for Youth Transitioning From Child to Adult Mental Health Services: Participatory Design Jam Study

**DOI:** 10.2196/91834

**Published:** 2026-05-29

**Authors:** Negar Vakili, Christine Cassidy, Sahil Chawla, Janet A Curran, Samantha Munro, Debbie Phillips, Ngoc Song Ha Pho, Roisin Walls, Alandra Wood, Lori Wozney

**Affiliations:** 1 Centre for Research in Family Health IWK Health Halifax, NS Canada; 2 School of Nursing Dalhousie University Halifax, NS Canada; 3 Faculty of Computer Science Dalhousie University Halifax, NS Canada; 4 Strengthening Transitions in Care Lab IWK Health Halifax, NS Canada; 5 Mental Health and Addictions IWK Health Halifax, NS Canada; 6 Mental Health and Addictions Nova Scotia Health Halifax, NS Canada

**Keywords:** text messaging, human-centered design, youth, transitions, technology-based interventions

## Abstract

**Background:**

The transition from child to adult mental health services is a vulnerable period marked by service disengagement, care gaps, and worsening mental health outcomes. Although planned, developmentally appropriate transition processes can improve functioning, youths report insufficient preparation, limited continuity of care, and unmet expectations for support. Existing transition supports remain underevaluated and require further adaptation for mental health contexts. Youth consistently report needing clearer information, concrete support, and sustained connection. Digital tools, particularly SMS text messaging, which is widely used, accessible, and acceptable to youth, offer a promising way to deliver timely transition supports. Yet most digital mental health tools are developed without meaningful youth involvement, highlighting the need for participatory approaches to ensure relevance, usability, and uptake.

**Objective:**

This study aimed to co-design and refine prototypes for a transition-focused SMS text messaging intervention by engaging youth with lived experience in a participatory co-design activity (design jam) to identify priority content, key functionality, and implementation enablers to support the transition from child to adult mental health services.

**Methods:**

We conducted a 3-hour mixed methods, participatory design jam to co-design transition-focused SMS text messaging prototypes, recruiting youth aged 16-26 years in Canada who had recently transitioned to, or were approaching transitioning to, adult mental health services. Data sources included workshop artifacts, observational field notes, and audio recordings from structured activities involving evidence review, brainstorming, rapid prototyping, brief team pitches, and evaluation. Rapid qualitative analysis, integrating open coding, content analysis of visual prototypes, and the rapid identification of themes from audio recordings, was used to identify priority content, key functionality, and implementation enablers. Findings were refined through a member-checking debrief with youth participants.

**Results:**

Seven youths aged 19-24 years participated in the design jam. Across two teams, participants generated 54 content ideas and 50 feature ideas. Two distinct prototypes were developed: one emphasizing long-term affirmation, self-advocacy, self-care, and profile-based customization, and the other prioritizing shorter-term informational support, navigation resources, and flexible message frequency. Youth across both groups highlighted the importance of interactive and visually engaging elements. Analysis revealed 3 thematic tensions shaping youth design preferences: balancing autonomy with ongoing support (roaming/reconnecting), balancing personalization with the need for simplicity (customization/convention), and balancing knowledge delivery with motivation for action (learning/living). Participants rated the design jam positively.

**Conclusions:**

Youth meaningfully contributed to co-designing an SMS text messaging intervention to support transition from child to adult mental health services, generating concrete content, functionality, and implementation priorities. Their prototypes highlighted the need to balance autonomy with support, personalization with simplicity, and information with motivational guidance. These findings demonstrate the value of participatory co-design in developing youth-centered digital transition supports and underscore the importance of evaluating such prototypes in real-world settings to determine feasibility and impact.

## Introduction

### Background and Rationale

The transition from child and adolescent mental health services (CAMHS) to adult mental health services (AMHS) typically occurs between the ages of 16 and 21 years. This period is particularly vulnerable, as youth tend to disengage from mental health services more frequently than other age groups [1,2]. Subsequently, youth often struggle to establish or maintain engagement with adult service providers [3], and when they do, they frequently present with more severe and enduring mental health problems [4]. Gaps and suboptimal care carry a risk of illness extension, progression, and chronicity, which have multiple adverse effects [5].

Young people who undergo a planned and purposeful transition process that addresses their psychosocial and medical needs as part of ongoing, coordinated care experience an improvement in their mental health and day-to-day functioning [2]. Even with a carefully planned transition, a young person's urge for autonomy and self-determination may influence their care trajectory [6,7]. Though many youths report having had conversations with their clinicians about transitioning to adult services, they expect more concrete support to ensure an appropriate transition actually takes place [8]. These complex developmental changes, coupled with policy gaps [9,10], contribute to few youths experiencing an “optimal transition” to AMHS. Different models of transitional mental health care for youth have been developed to maintain continuity of care. However, systematic reviews reveal a lack of adequately powered studies, randomized controlled trials, or case-controlled studies evaluating their effectiveness [9]. While recommendations have been developed to support general transitions in care for youth with chronic or complex illnesses [11], there is still a need to tailor how those recommendations are put into action for mental health contexts specifically [12].

Previous qualitative research with youth suggests several key enablers for effective transitions: informal and gradual preparation for transition, accessible and developmentally appropriate services on the adult side, transfer planning meetings, periods of parallel care, consistency in key clinicians, caregiver involvement, and individualized wrap-around services within the community [13-15]. Youth value time for preparation, flexible timing, personalized plans, information continuity, collaborative efforts, and relational continuity [6]. Given the vulnerability of this period and accumulating evidence around what youth prefer and need, it is crucial to rapidly translate this evidence into testable interventions that can be implemented into care pathways.

### Health Technology and Participatory Design

Technology-based services for youth transitioning to AMHS have been shown to be both cost-effective and clinically effective in other chronic illness contexts (eg, diabetes, cystic fibrosis, and bowel disease) [16,17]. Youth transitioning to AMHS may benefit from modern technology modalities that align with their preferences and reflect how they use digital tools every day [18-20]. Text messaging, also known as short-messaging service (SMS), is among the most frequently used technologies for delivering low-intensity behavioral health interventions and is acceptable, scalable, and has been used successfully across a range of adolescent mental health service contexts [21]. Drawing on emerging insights into human-computer interaction and persuasive system design features [22,23], an SMS text message intervention could engage youth in ways that traditional paper-based or in-person services cannot [18]. This approach offers ease of access, organized content, personalization, and cost-effective implementation, making it an optimal choice for engaging youth [24]. Historically, the design process in health technologies has relied on top-down approaches that can be characterized as “technocentrically oriented” [25]. The patient or end user is conceptualized as a “human factor”—rather than a real partner in the design process [26]. If we are to humanize digital mental health interventions that might support youth transitioning to AMHS, researchers and designers need to use collective design methods to elicit diverse ideas. The involvement of people with lived experience in this way can enhance intervention effectiveness and increase the likelihood of system adoption [27]. Accordingly, the aim of this study was to engage youth with lived experience in a participatory design jam to co-design the core content, key functionality, and implementation considerations for an SMS text messaging intervention that supports the transition from CAMHS to AMHS.

## Methods

### Study Design

Our mixed methods participatory approach resulted in data collected from 3 sources: observational field notes, audio recordings, and artifacts including large posters, sticky notes, and photos. The study involved engaging youth in a design jam. Design jams are prototype-driven, as prototype creation is seen as an activity to better understand, explore, and communicate fuzzy, early ideas, and get a sense of what it could be like to interact with such a solution in real life [28-31]. Design jams bring individuals from different perspectives together to build empathy, think by doing, visualize ideas, combine divergent and convergent approaches, and foster collaboration and empowerment among potential beneficiaries [32]. This approach is aligned with extant literature on rapid prototype development within the context of human-centered design, where researchers and practitioners seek to iterate and “fail fast to succeed sooner” in their iteration of possible solutions [33-35].

### Setting, Population, and Recruitment

Youth were eligible to participate if they met the following basic criteria: (1) youth living in Canada (ages 16-26 years) who had transitioned to AMHS within the last 5 years or would transition within the next 5 years, (2) able to communicate in English, and (3) able to attend the workshop in person. Design jams vary in size but typically involve teams with fewer than 10 people [29]. We used a multichannel recruitment strategy, including social media, professional networks of study collaborators, newsletters, and distribution lists of partner organizations. Once contact was initiated by potential participants, the study coordinator emailed the consent form and scheduled a phone call to review and answer questions. The study was conducted in 2023 in Canada.

### Design Jam Procedure

The design jam procedures for this study were informed by feedback from youth with lived experience and the broader research team. The goal of the 3-hour design jam was to (1) establish priority topics and content for SMS text messages that would be sent in a future intervention, (2) prioritize key technical and functional aspects of the intervention (eg, number of texts to be sent, timing, and frequency), and (3) generate ideas for enablers that could help overcome barriers to uptake and implementation of an SMS text message–based intervention within community mental health services.

The design jam was held on a weekend from 10 AM to 1 PM to ensure youth participants were not in school. It was held in a collaborative workspace at a public community library for easier transportation accessibility. The event started with a short icebreaker to build rapport between participants and facilitators and stimulate creative thinking. The group was split into two teams (HEARTS and SPADES). Three facilitators (NV, LW, and SP) worked to support activities and took jot notes and pictures throughout the day. Artifacts generated or modified by participants during the workshop, such as poster board notes, color-coded stickies, and dot voting (a simple prioritization technique in which participants place adhesive dots beside ideas they prefer), provided valuable resources for later analysis [36]. Digital audio recordings were made during the 5 key activities: reviewing previous evidence, brainstorming, prototyping, pitching, and evaluation sessions.

### Activity 1: Reviewing Previous Evidence

The first activity was to familiarize participants with key evidence from prior work. We reviewed the findings of Vakili et al [18], where 100 youths provided their perspectives on critical items to support the transition to AMHS. We presented a list of priority content and functionality items from that study, which had a high level of consensus regarding their importance. For example, description of what to expect in their first mental health appointment; contact information for individuals youth can consult if they have questions; affirmation of feelings of frustration or disappointment during the change and reminder of available self-care support; information tailored to specific mental health conditions; the ability to stop/start texts at any point; information that sounds truthful, fair, and unbiased; and evidence that texts were coming from a trusted health care center. Finally, we reviewed the most significant enablers (eg, personalized messages, messages that captured their attention, and receiving a reasonable number of texts) and barriers (eg, messages being too long or too many) identified in the prior study. Design jam participants were prompted to consider these priorities during their prototyping activity.

### Activity 2: Individual and Group Brainstorming

**Figure 1:**
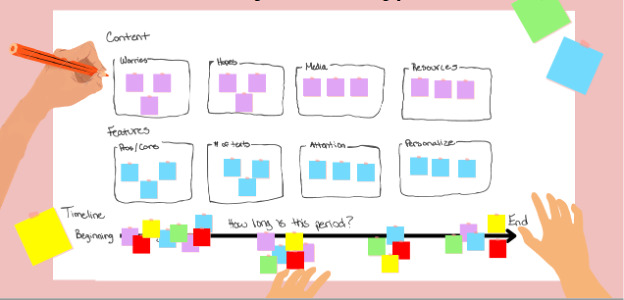
Design canvas.

**Figure 2:**
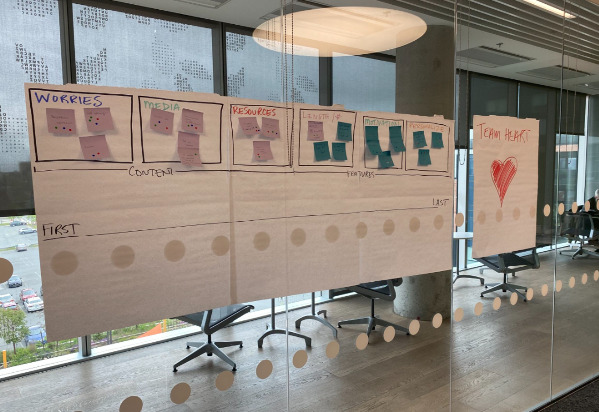
Team HEARTS' initial design canvas.

Both teams were introduced to the concept of a “design canvas” (see Figure 1) as an organizing framework to facilitate rapid prototyping. Completing the design canvas began with rapid rounds of individual and group brainstorming. Each brainstorming round followed a timed cycle of (1) individually writing down as many ideas as possible on sticky notes [2 minutes]; (2) affixing all notes to the wall and, as a group, reviewing and categorizing those ideas [2 minutes]; and (3) using dot voting to allow each participant to review and select their top 3 choices [2 minutes] (see Figure 2). To brainstorm the preferred *content* of the messages, youth were prompted to write out what worried them about the transition to adult services, what they hoped would come of the transition process, the types of media they preferred to engage with within a text (eg, video clips and images), and any helpful external resources they believed would be key for the intervention to direct youth toward. To brainstorm the preferred *functionality*, youth were prompted to write out the total number of texts that should comprise the intervention, the duration of time over which youth would receive these texts, strategies to maintain youth engagement with the texts, and methods to personalize the texts to enhance their relevance.

### Activity 3: Prototyping

Each team’s top 3 choices from the brainstorming sessions, identified through dot voting, were retained on the canvas to support prototype building, ensuring that the most valued ideas were prioritized. Participants then worked as a team to refine and develop a concept and chronology for their intervention prototype. They moved sticky notes to reorganize ideas, used markers and stickers to add details and annotations, and organized the information into a coherent timeline. These activities facilitated a dynamic and interactive environment where participants could visualize and iterate on their ideas together. Group discussions and problem-solving were integral to the process, providing opportunities for participants to share their thoughts and build off of each other’s ideas. A facilitator was present to guide the discussions and clarify questions about the activity as needed, ensuring that the prototyping moved forward in a timely manner.

### Activity 4: Pitching

**Figure 3:**
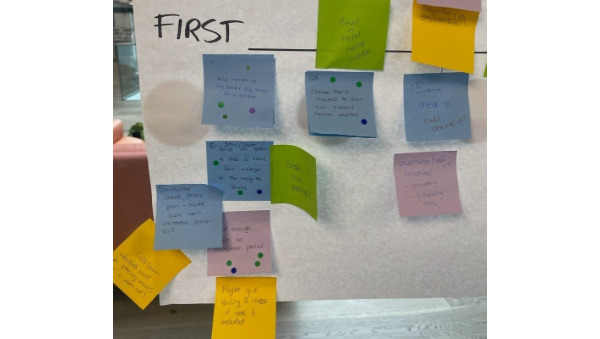
Team SPADES' prototype pitching feedback.

Following a brief intermission, members from each team were given the opportunity to prepare a concise, under-5-minute pitch for their prototype that would be presented to the full group. In this way, youth participated in peer-to-peer feedback, constructive dialogue about the pros and cons of each prototype concept, and suggestions to further strengthen great ideas (see Figure 3). To facilitate this process, colored sticky notes were given to everyone after each pitch to add to the canvas: green for great ideas, red for less favorable ideas, and yellow for uncertain ideas. This method served as an effective tool for group prioritization and prototype refinement (see Figure 3). Lastly, a facilitated large group discussion was held as a form of analytic dialogue to collaboratively discuss the commonalities and differences between the two prototypes and how the final designs resonated—or failed to resonate—with the group.

### Activity 5: Evaluation

Structured feedback opportunities for design jams are highly recommended [37]. This exercise serves a dual purpose: it acts as an informal evaluation of the event's successes and areas needing improvement, and it reinforces the significance of youth research engagement and their role in the co-design process. To gather feedback, a brief questionnaire asked participants to rate their overall experience in the design jam on a scale from 1 (very poor) to 5 (excellent). Additionally, 2 open-ended questions were included: “What would have made your experience better?” and “What was your favorite part of the day?”

### Analysis

The goal of the analysis was to distill both the explicit content and functionality features of the two prototypes and uncover implicit tensions, considerations, or complexities not identified in earlier phases of research that might impact the real-world rollout of an intervention within a clinical service. Rapid analysis of qualitative data is a key component of the human-centered design methodology, as it plays a role in swift transitions between information gathering and solution development/testing. However, the use of these methods recognizes the inherent tension between speed, rigor, and collaborative meaning-making [38]. We followed guidance on combining multiple analytic methods to optimize evidential utility [39], including (1) open coding of facilitator field notes, (2) content analysis of visual prototypes and event photos, and (3) rapid identification of themes from audio recordings [40]. Taken together, this approach provided completeness (timeline photos), kept the data fresh for analysis (recording), and ensured grounding in context (field notes).

As a first step, the senior researcher (LW) reviewed and tabulated the results of dot voting activities and themed field notes, and performed preliminary content analysis of photos of the prototypes. In the second step, 3 authors (LW, NV, and AW) listened to audio recordings, facilitating a second layer of analysis and extracting additional contextual information. To reduce researcher bias, promote triangulation in interpretation, and adhere to the principles of co-design, a third step involved a data debrief session [41] with 4 of the design jam participants and a peer researcher. The 1-hour session was conducted using a structured meeting guide that aimed to spark reflection on emerging findings, better position findings, identify gaps and limitations, seek out alternative perspectives (such as negative or disconfirming cases), and add or modify key discussion points.

### Ethical Considerations

This study was approved by the IWK Health Centre Research Ethics Board (REB #1027212). Informed consent was obtained from all participants prior to attending the design jam. Participation in the design jam was voluntary, and participants received a CAD $100 (approximately US $72.96) gift card honorarium. All information gathered about participants was kept private and confidential.

## Results

### Demographics

There were 16 potential participants who initially responded to the recruitment opportunity. However, due to scheduling conflicts and unexpected personal and work demands, only 7 participants between the ages of 19 and 24 were able to attend. Overall, 86% (6/7) of participants identified as women. Moreover, 2 participants identified as members of the 2SLGBTQIA+ community and 2 as persons who are differently abled (eg, visual impairment, learning disability, and speech impairment). Participants self-identified with White, South Asian, and East Asian racial groups. Additionally, 71% (5/7) were housed and living on their own. All participants were either currently enrolled in or had graduated from a postsecondary institution. The duration between when participants first accessed mental health services and the date of the design jam ranged from 4 to 12 years.

### Content and Functionality of Prototypes

Initial brainstorming generated 54 unique content ideas (eg, worries, media, and resources) and 50 unique feature ideas (eg, length, motivation, and personalization) across both groups. The results of the dot-voting prioritization are reported in Table 1.

**Table 1:** Top 3 priorities based on dot voting for key content and features.

Key areas	Team HEARTS	Team SPADES
Worries	New/different expectations Self-advocacy Expenses	Not enough info on transition period (what to expect) Not knowing where to go (what resources are available and how to access them) Wait times (having to wait months between appointments)
Media	Contact info for services and supports Calm/neutral colors Self-care reminders (eg, journal for self-care and to-do lists)	Video/presentation of stories from other youth who transitioned from CAMHS^a^ to AMHS^b^ Interactive graphics (maps, animations, quizzes) Exercises (eg, breathing and how to stop grinding teeth)
Resources	A wellness map^c^ Push dates for the mental health services (ie, reminder for appointment) List of professionals who work with younger people	Low income (employment, free programs and services) Self-help mobile apps Interest groups
Length	12 months with 3- and 6-month check-ins 7 AM texts or evening after dinner time Daily	3-6 months with a 3-month check-in 2-3 texts per week, spaced throughout Slowly reduce frequency over time
Motivation	Cute pictures/memes/stickers Mini quizzes and check-ins with badges Short quotes or affirmations	Use informal language (keeping it chill) Self-check quiz Questions in the texts that you want to answer/respond to
Personalization	Set up personal profile Ability to modify profile Drilling down into relevant topics (ie, increase texts related to housing if that was of interest)	Refer to user by their first name Option to stop/start and take breaks from getting the messages Choose topics related to your specific mental health condition (specific to mental illness or type of care needed)

^a^CAMHS: child and adolescent mental health services.

^b^AMHS: adult mental health services.

^c^A self-developed transition wellness app that offers resources, reminders, and a journal for writing.

**Figure 4:**
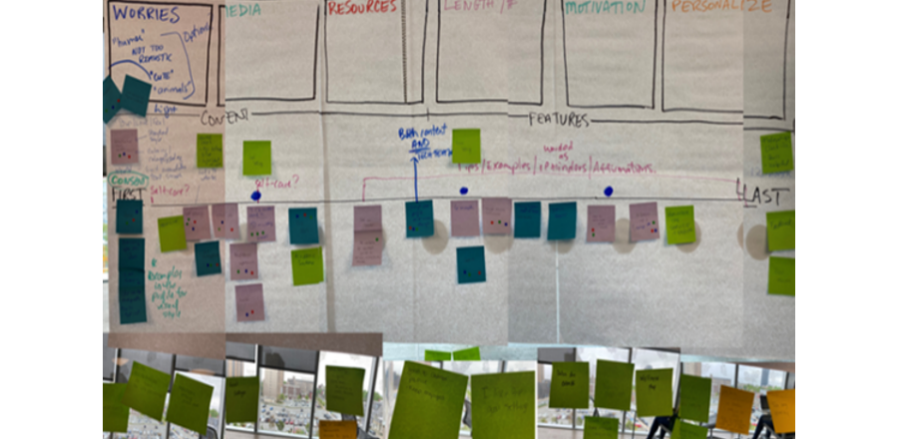
Prototype 1: Team HEARTS.

**Figure 5:**
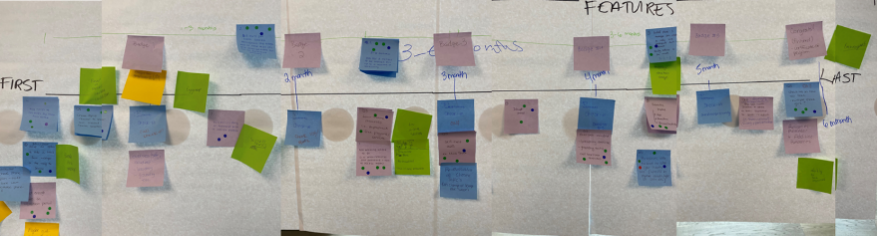
Prototype 2: Team SPADES.

While there were some overlapping key priorities identified (ie, use of self-check-in quizzes, contact information and navigation support for services, and ability to modify some aspects of the messaging service content), the prototypes diverged on many aspects. For example, one team recommended a 12-month service with daily SMS text messages and a self-check-in assessment every 3 months, whereas the other team proposed a shorter 3-6-month intervention with texts sent every 2-3 days. Regardless of the length and frequency, there was an overall emphasis on interactive elements (eg, interactive graphics, quizzes, ability to drill down on topics, and creating a personal profile) and visual components (eg, colors, videos, memes, stickers, and cute photos). Figures 4 and 5 illustrate the two prototypes.

Team HEARTS produced a prototype that generally focused on *affirmation and encouragement over the long term*. Participants suggested (1) *self-care*, (2) *exploration* (eg, journaling and drilling down on topics of interest), and (3) *self-advocacy* as priority ways the SMS text messaging intervention could support the transition to adult services. Besides the ability to set up and choose topics of interest through a personal profile—which would determine which texts were sent on which topics—it was recognized that the intervention would also need to attend to the motivational needs of youth to ensure long-term engagement. A suggestion for improving engagement was to use short affirming messages that conveyed warmth and humor, along with providing links to supplemental resources. Additionally, incorporating calming and neutral colors for the intervention elements was recommended. During the pitch session, feedback on the personal profile set-up led to additional recommendations for a consent process for data, the use of an organizational logo to enhance credibility, and ensuring there is no cost for youth to sign up. One participant added that if the intervention were to offer the ability to “drill down” on topics, the information provided should be evidence- or science-based. A red flag was raised during the pitch session about the length of the intervention and the insufficient number of check-ins to keep youth engaged.

Team SPADES produced a prototype that primarily focused on *information and navigation support*. Participants suggested three key areas: (1) *connection to local resources* (eg, links to interest groups, contact information for professionals, and links to free programs and services), (2) *expectation management* (eg, understanding what to anticipate during the transition period), and (3) *information tailored to specific mental health conditions*. The prototype priorities for functionality also reflected an emphasis on youth choice and autonomy. This included managing the intensity of the intervention by gradually reducing the frequency of SMS text messages over time. For instance, starting with 3 messages per week to maintain participant interest, then slowly reducing to 1 message per week. The aim was to find a balance where the frequency was “not so little that you lose engagement, but not so frequent that it becomes overwhelming.” Feedback from the pitch session noted that while the use of badges “might feel a little gamey/superficial,” offering congratulations on milestone achievements would be a positive way to encourage youth. Additionally, allowing participants to take breaks from receiving SMS text messages was seen as a beneficial approach to maintaining their engagement.

### Key Themes

Three thematic tensions emerged during data analysis: roaming/reconnecting, learning/living, and customization/convention. These dimensions encapsulate the inherent trade-offs and challenges that youth spoke about in trying to design an intervention prototype that addresses the complex psychosocial needs of youth at a complex time in their lives.

#### Roaming and Reconnecting: Focus on Taking Responsibility and Exploring Your Own Needs Versus Needing More Direct Adult Guidance and Help

Youth reflected on how autonomy is an essential component of the transition process, but total independence may not always be beneficial. They acknowledged that they may initially lack the maturity, experience, or coping mechanisms needed to navigate complex situations without adequate guidance. The SMS text message intervention would be best able to support them if it gradually encouraged them to take on more responsibility as they developed the skills and confidence necessary for managing their mental health care as an adult. One participant stated,

I feel...like either there should be at all times...able to stop the messages but maybe do a formal check-in at every 3 months, 6 months and see where people are at. Because I mean if somebody wants it for a year we shouldn’t want to take it away from them but if they need it like, I know for me I prefer the whole like hand-holding throughout the whole thing but if someone isn’t like me and doesn’t need the hand-holding and they’re cool after 3 months or so or 6 months then...

Participant #2, female

Team HEARTS stressed the significance of “Setting up a profile page where you actually get to identify things that you want and it automates it based on your characteristics.” This issue was also reflected in Team HEARTS’ focus on promoting skills in self-advocacy, with one of the participating youth remarking, “Self-advocacy is something I would like to [learn about] right away” in the intervention. At the same time, Team HEARTS also emphasized the need to reinforce connection and a sense of not being alone on the journey. This manifested in the prototype as early sharing of contact information for crisis resources, such as helplines, and the value of daily affirmations that involved “cute” pictures or simple messages of “You did good today!” to provide positive reinforcement and encouragement.

Members of Team SPADES shared strong support for the intervention helping youth exercise their autonomy and develop necessary skills through connection to a network of supports in the community. Rather than reminders of urgent care options, they saw value in providing a comprehensive array of resources, including low-income and employment resources, free services in the community, locations of mental health services in their neighborhood, self-help applications, information on local interest groups, and interactive graphics. One participating youth highlighted the importance of this approach, stating, “low-income resources, I think, [are] super important.” The recommendation for a monthly reward “badge” system and end-of-intervention certificate also speaks to this view that external motivators were important for the intervention to incorporate. This “badge” system exemplifies a self-monitoring strategy commonly found in persuasive system designs, encouraging users to complete tasks and maintain progress [42]. Reducing the frequency of texts over time but having frequent initial messages also reflected this notion of graduated independence.

#### Customization and Convention: Ability to Deeply Personalize the Intervention Versus the Necessity of Simplicity

Team HEARTS put significant emphasis on integrating profile-based check-ins. These check-ins would allow users to modify their personal profiles, desired topic areas, and frequency of messages. One youth suggested a prompt asking, “[Is] there anything you want to change?...Because sometimes over the year you might be like depressed but then all of a sudden you have something else that’s a different problem.” Ideally, this would offer youth the autonomy to adjust aspects of the intervention they liked or disliked, reflecting their evolving experience and needs. During Team HEARTS’ pitch, they emphasized this, saying,

...We kind of wanted to give autonomy for individuals to select that with their profile just because some individuals might feel like they want a daily text message, but other people might feel like that’s a bit overbearing and overwhelming, so we wanted to give kind of the individual the ability to choose what would be helpful.

Participant #6, female

However, as pointed out in the peer-feedback portion of the pitch event, creating these profiles can add to cognitive load. In addition, the level of data entry needed to generate a profile might result in a click-heavy, data-intensive, and burdensome process linked to poor user experience.

Team SPADES concurred with some aspects of customization proposed by Team HEARTS, such as the ability to reevaluate the choice of topics. For example, if users initially chose to learn about how to manage anxiety during the transition but later changed their minds, they would have the ability to reset preferences at timed intervals. However, Team SPADES’ prototype had a simpler design, over a shorter duration, with less contact overall. These differences point to the challenge of balancing a simple automated process, choice, and more importantly, the need for those choices to be modifiable over time.

#### Learning and Living: Focus on Acquiring New Knowledge Versus Focus on Motivating Action

The fact that over 50 unique content ideas were generated by participating youth in under 2 minutes of rapid brainstorming speaks to the wide range of informational needs youth perceive as relevant during the transition period. At the same time, youth also recognized that acquiring more knowledge was not the only function the SMS text messages could serve. Importantly, youth saw value in the texts offering motivational support needed to take on the actions necessary to connect with the AMHS in ways that would benefit their mental health journey. One of the participating youth quoted,

...Motivational videos would be good. What I was actually thinking [was] like increasing and telling the participants you just have 1 month left, and you are going to do it. Just giving that moral support.

Participant #3, male

Team SPADES prioritized new knowledge acquisition in their prototype. Addressing information gaps regarding the transition period was a primary concern. To mitigate this, they proposed an initial focus on providing comprehensive information about the transition. Moreover, they suggested introducing new information at monthly intervals to avoid cognitive overload and allow youth to absorb the information received before introducing further content. One youth remarked,

...Sometimes when I’m trying to figure something out, look at resources and understand what’s going on, its already entirely overwhelming and stressful and you don’t really know where you’re going and bombarded by a bunch of different information, so the more we can honestly just like address that...I feel like the better.

Participant #6, female

To enhance the motivational impact of an SMS text message intervention, Team SPADES proposed the use of success stories from other youth who have transitioned to AMHS. Linking within an SMS text message to a video clip or short narrative from another youth was positioned as a way to inspire, model, and bring a sense of hope that taking positive actions could lead to a better transition experience.

Team HEARTS identified moving from knowledge to skills practice as a beneficial preparatory activity for individuals undergoing transitions, and one that the intervention could support. For example, one suggestion was to offer an option for a downloadable journal through the texts. The journal could provide practical exercises and reflections, thereby supporting the development of effective self-care practices. Both teams highlighted the concept of “check-ins” or regular points in the intervention timeline when youth could complete a self-assessment quiz, be prompted to reflect, check in on their progress, and think ahead to what they wanted to focus on next. In this way, the SMS text message intervention would need to play a dual focus of delivering informational content while also attending to sustain motivation in new ways. As one participant noted: “I like that we’re sort of saying yeah there’s content and features, but there’s also this piece that’s about look and feel, the kind of vibe of the messages.”

### Design Jam Evaluation

The design jam was well-received by participating youth, who found the workshop activities and structures to be both creative and collaborative. One participant stated, “My favorite part was the pitch. It was so great to hear everyone’s thoughts and ideas presented as one.” Another participant shared similar feedback, noting that “It allowed for open discussion surrounding differences and similarities between the timelines, giving a ton of fantastic ideas.” However, participants also suggested improvements, such as increasing the diversity among attendees and allowing more time to process information. Most rated their overall experience of the Jam as “excellent,” with one youth rating the experience between “good” and “excellent.”

## Discussion

### Principal Findings

This study aimed to engage youth with lived experiences in a participatory design jam to co-design core content, functionality, and implementation considerations for an SMS text message intervention supporting transitions from CAMHS to AMHS. Youth successfully produced two distinct low-fidelity prototypes and prioritized interactive, personalized features alongside implementation enablers and barriers. Three crosscutting tensions, roaming/reconnecting, customization/convention, and learning/living, surfaced as central design considerations.

While most research on youth transition needs and preferences focuses on prioritization, our study identified how youth would structure an intervention to best meet their needs. Despite some participants’ unfamiliarity with design jams or SMS text messaging interventions, the youth participants successfully designed basic prototypes, including core content, core functionality, and identified barriers and enablers to adoption [43]. The successful generation of these prototypes and the diversity observed among just 7 participants in 2 groups demonstrate the potential of involving youth in more creative design solution work within the digital mental health space.

In the context of health interventions, customization is often viewed as critical, as individuals encounter health issues differently based on their unique cultural, social, and psychological backgrounds [44]. Findings from this study support previous work demonstrating that transitioning to adulthood often requires evolving interventions that can keep up with the changing cognitive, emotional, and social needs of youth. Findings also strongly support the idea that providing youth with structured guidance and support systems during this period, including mentorship, skill-building programs, and access to community resources [45,46], are important enablers. At the same time, youth emphasized that personalization must be balanced with usability, particularly in SMS, where brevity can conflict with information needs, suggesting periodic preference reset points as a pragmatic way to maintain fit over time. However, developing tailored solutions can complicate the design process. Highly personalized features may add technological complexity, making navigation more difficult for users [47]. SMS text messages, which are typically limited to 160 characters [48], may be a challenging medium for finding a balance between oversimplification that prioritizes ease of use and complex, information-dense materials that may lead to disengagement. Achieving an optimal balance between personalization and ease of use presents a weighty obstacle for designers and developers [49]. Youth in this study had a clear suggestion that the ability to adjust preferences at regular intervals within the SMS text message intervention was a vital design component, one that had not been identified in earlier studies.

This participatory approach fosters a sense of ownership and empowerment among youth, ultimately leading to better health outcomes and more successful transitions to adult services [27]. In participatory design, patient engagement is crucial and must be encouraged through various methods, including talking, doing, making, and enacting. Engaging youth in the design process ensures that the intervention is relevant and user-friendly. This approach requires robust real-world community evaluations to understand the potential for wide-scale implementations. These stages are necessary to contribute to the development of a theory of change that could explain and justify how co-design research methods lead to better health outcomes [18].

### Strengths and Limitations

A key strength of this study is the involvement of youth with lived experience throughout and the creative approach taken during the design process. Both teams at the design jam successfully created a prototype. However, there are several important limitations to consider. The funding for this project steered the co-design process toward developing an SMS text message–based intervention. This constrained the ability to critically assess whether an SMS text message solution was truly the most appropriate. Additionally, the results regarding ideas and preferences cannot be universally applied to the entire youth population. The sample size (n=7) and the use of only two groups limit generalizability and may have amplified group-specific preferences. There may be a self-selection bias as participants volunteered to take part in the design jam, particularly if youth with prior knowledge or interest in health technologies attended the workshop. Young people without such interests might have had different preferences or ideas. Furthermore, it is likely that the tasks and facilitation styles throughout the workshop influenced the final prototypes. For example, time-boxed brainstorming cycles, the use of dot voting to prioritize ideas, and facilitator prompts to keep activities moving may have nudged participants toward concise, easily implementable features and away from more complex or slower-to-develop concepts. Finally, the single-session format precluded iterative testing; multisession cycles could yield different or more refined suggestions.

### Future Direction and Recommendations

Building on the findings from this design jam, future work should first translate the youth-generated prototypes and the 3 thematic tensions (roaming/reconnecting, customization/convention, and learning/living) into a testable minimum viable SMS intervention. This includes operationalizing key youth-identified features such as scheduled check-ins (eg, at 3 and 6 months), customized message frequency, lightweight personalization options, and pairing informational content with motivational prompts. Future efforts should focus on feasibility and usability testing in real-world settings (eg, engagement, acceptability, burden, and continuity-of-care/transition follow-through) to determine whether these youth-identified features meaningfully support transition readiness. Subsequent pragmatic evaluations can examine how this SMS intervention integrates with existing transition models [9], how tailoring to individual needs can be implemented without adding excessive complexity [9], and how policy frameworks can enable scalable, no-cost access and credible cobranding within services [9,10]. By grounding next steps in what youth themselves prioritized, and iteratively testing these concrete features, future work can move beyond concept generation toward scalable interventions that improve transition experience and well-being.

### Conclusions

Participatory co-design with youth is not only feasible but also productive for translating high-level transition principles into concrete digital design requirements and implementation hypotheses. These 3 identified tensions offer a practical framework for specifying features (eg, scheduled preference resets, message frequency, and integrated motivational prompts) that service partners can trial in real-world pathways. The design jam approach provided valuable insights into the development of a low-fidelity prototype to support the transition of youth from CAMHS to AMHS. Youth involvement in the design process, despite their initial unfamiliarity, resulted in practical and varied prototypes that address core content, core functionality, and barriers to adoption [43]. Overall, by addressing the identified experiential tensions and leveraging participatory design methods, systems can develop more effective and user-centered interventions to support youth during this critical transition period.

## Abbreviations

AMHS: adult mental health services
CAMHS: child and adolescent mental health services

## Data Availability

The datasets generated and/or analyzed during this study are available from the corresponding author on reasonable request.
